# Modulation of interleukin-1β-induced inflammatory responses by a synthetic cationic innate defence regulator peptide, IDR-1002, in synovial fibroblasts

**DOI:** 10.1186/ar3440

**Published:** 2011-08-11

**Authors:** Emily Turner-Brannen, Ka-Yee Choi, Dustin ND Lippert, John P Cortens, Robert EW Hancock, Hani El-Gabalawy, Neeloffer Mookherjee

**Affiliations:** 1 Manitoba Centre for Proteomics and Systems Biology, Department of Internal Medicine, University of Manitoba, 799 John Buhler Research Centre, 715 McDermot Avenue, Winnipeg, MB, R3E3P4, Canada; 2 Department of Immunology, University of Manitoba, 471 Apotex Centre, 750 McDermot Avenue, Winnipeg, MB, R3E0T5, Canada; 3 Centre for Microbial Diseases and Immunity Research, University of British Columbia, 2259 Lower Mall Research Station, Vancouver, BC, V6T1Z4, Canada

## Abstract

**Introduction:**

Innate defence regulator (IDR) peptides are synthetic cationic peptides, variants of naturally occurring innate immune effector molecules known as host defence peptides. IDR peptides were recently demonstrated to limit infection-associated inflammation selectively without compromising host innate immune functions. This study examined the impact of a 12-amino acid IDR peptide, IDR-1002, in pro-inflammatory cytokine interleukin (IL)-1β-induced responses in synovial fibroblasts, a critical cell type in the pathogenesis of inflammatory arthritis.

**Methods:**

Human fibroblast-like synoviocytes (FLS) were stimulated with IL-1β in the presence and absence of IDR-1002. Production of enzyme matrix metalloproteinase-3 (MMP-3) and IL-1-receptor antagonist (IL-1RA) was monitored by enzyme-linked immunosorbent assay (ELISA), and various chemokines were evaluated by using multiplex cytometric bead array. Transcriptional responses were analyzed by quantitative real-time PCR. The impact on IL-1β-induced proteome was investigated by quantitative proteomics by using isobaric tags. IL-1β-induced pathways altered by IDR-1002 implicated by the proteomics analyses were further investigated by using various immunochemical assays. Cellular uptake of the peptide was monitored by using a biotinylated IDR-1002 peptide followed by microscopy probing with streptavidin-Alexa Fluor.

**Results:**

This study demonstrated that IDR-1002 suppressed the production of IL-1β-induced MMP-3 and monocyte chemotactic protein-1 (MCP-1); in contrast, IDR-1002 enhanced the production of IL-1RA, without neutralizing all chemokine responses. IDR-1002 altered the IL-1β-induced proteome primarily by altering the expression of members of nuclear factor kappa-B (NF-κB) and c-Jun N-terminal kinase (JNK) pathways. The proteomics data also suggested that IDR-1002 was altering the transcription factor HNF-4α-mediated responses, known to be critical in metabolic regulation. With various immunochemical assays, it was further demonstrated that IL-1β-induced NF-κB, JNK, and p38 mitogen-activated protein kinase (MAPK) activations were significantly suppressed by IDR-1002.

**Conclusions:**

This study demonstrates the ability of an innate immune-modulatory IDR-peptide to influence the IL-1β-induced regulatory pathways and selectively to suppress inflammatory responses in synovial fibroblasts. The results of this study provide a rationale for examining the use of IDR-peptides as potential therapeutic candidates for chronic inflammatory diseases such as inflammatory arthritis.

## Introduction

Cationic host defense peptides (HDPs) are naturally occurring effector molecules of innate immunity. These peptides are 12 to 50 amino acids in length, with a net positive charge ranging from +2 to +7 with up to 50% hydrophobic amino acids [1]. HDPs exhibit a wide variety of immunomodulatory functions and delicately modulate inflammatory responses without compromising the elements of immunity required for resolution of infections [2-8]. HDPs exhibit anti-inflammatory effects by suppressing certain pro-inflammatory pathways, upregulating anti-inflammatory mechanisms (for example, IL-10), and intervening in the activation of nuclear factor (NF)-κB via multiple mechanisms [3]. A broad spectrum of cationic HDPs are expressed in human synovium tissues with differential expression patterns under inflammatory conditions [9]. However, the role of HDPs in synovium biology is not well characterized. It has been suggested that induction of HDPs by vitamin D may play a role in the protection against autoimmune diseases such as rheumatoid arthritis (RA) [10]. Therefore, HDPs and their derivatives are attractive candidates for modulating the inflammatory responses in chronic inflammatory disorders, including in inflammatory arthritis.

HDPs are widely diverse in sequence and structure, and this wide repertoire provides an extensive template for designing short synthetic peptides with optimized activities and reduced cytotoxicities [11-13]. The synthetic variants of HDP are known as innate defence regulator (IDR) peptides [14]. Two IDR peptides, IDR-1 and IDR-1002, have been shown to protect against infections largely by modulating innate immune responses of the host and upregulating anti-inflammatory mechanisms [15,16]. To our knowledge, no studies to date have investigated the potential of IDR peptides in limiting inflammation in immune-mediated chronic inflammatory disorders such as inflammatory arthritis.

The complex pathophysiology of arthritis involves synergistic interplay primarily between mesenchymal cells such as fibroblast-like synoviocytes (FLS) and immune cells (for example, macrophages and T-lymphocytes). Activation of FLS by pro-inflammatory cytokines results in the production of inflammatory cytokines, chemokines, and matrix-degrading matrix metallopeptidases (MMPs), which lead to the destruction of articular cartilage and bone [17]. TNF-α and IL-1β are two inflammatory cytokines that are well defined as critical inflammatory mediators in arthritis [18]. TNF-α is proposed to be the dominant pro-inflammatory cytokine in the inflammatory manifestations of synovitis, whereas IL-1β is thought to be important in the destructive potential of chronic joint inflammation [19,20]. IL-1β induces the production of MMP-3 in cell types such as FLS, chondrocytes, and macrophages in arthritic joints, and the subsequent elevated level of MMP-3 mediates cartilage and bone destruction, directly contributing to the pathogenesis of the disease [21]. Efficacious therapeutic strategies have been developed to target each of these cytokines. Despite this, a major consideration regarding therapeutic agents targeting inflammatory cytokines such as TNF-α is the increased associated risk of infections and neoplasm [22,23]. This highlights the need for the development of alternate strategies for the management of chronic inflammatory arthropathies. We hypothesized that one such strategy would be to examine the use of selectively immune-modulatory agents such as IDR peptides [24].

In this study, we demonstrated that a 12-amino-acid IDR peptide, IDR-1002 [16], suppressed IL-1β-mediated cellular responses in human FLS, especially MMP-3 and MCP-1 production. However, IDR-1002 did not neutralize all IL-1β-induced chemokine responses that are required for resolution of infections. In contrast, this peptide enhanced the production of negative regulators of IL-1β (for example, IL-1-receptor antagonist (IL-1RA). These observations were consistent with the paradigm of the selective anti-inflammatory mechanism of host defense and IDR peptides [3,15,16]. We explored the molecular mechanism of regulation of IL-1β-induced responses by IDR-1002 in FLSs, by using quantitative proteomics and other immunochemical assays. We demonstrated that IDR-1002 suppressed IL-1β-induced NF-κB, c-Jun kinase (JNK), and p38 mitogen-activated protein kinase (MAPK) activation in synovial fibroblasts. This study provides a rationale for further examining the use of IDR peptides as potential therapeutics for the management of inflammatory arthritis and possibly other diseases characterized by chronic inflammation.

## Materials and methods

### Cell isolation and culture

Synovial tissues were obtained from patients with osteoarthritis (OA) or rheumatoid arthritis (RA) with informed consent in accordance with a protocol approved by the Institutional Review Board at the University of Manitoba. Human FLS were isolated from the synovial tissues, as previously described [25]. In brief, the tissues were digested with 1 mg/ml collagenase and 0.05 mg/ml hyaluronidase (Sigma Aldrich) in Hanks' balanced salt solution (Gibco; Invitrogen Inc., Burlington, ON, Canada) for 1 to 2 hours at 37°C. Cells were washed and cultured in DMEM media (Gibco), supplemented with sodium pyruvate and nonessential amino acids (referred to as complete DMEM media henceforth) containing 10% (vol/vol) fetal bovine serum (FBS) in a humidified incubator at 37°C and 10% CO_2_. Isolated human FLS (*ex vivo) *were seeded at 2 × 10^4 ^cells/ml, either 0.5 ml per well in 48-well tissue-culture plate, or 3 ml per well in six-well tissue-culture plate, as required, and cultured in complete DMEM media containing 10% (vol/vol) FBS overnight. The following day, the culture media was changed to complete DMEM containing 1% (vol/vol) FBS before the addition of the various stimulants. A rabbit synoviocyte cell line HIG-82 (ATCC CRL-1832) was cultured in Ham's F-12 growth medium containing glutamine (Gibco) supplemented with sodium pyruvate (referred to as complete F-12 media henceforth), containing 10% (vol/vol) FBS in a humidified incubator at 37°C and 5% CO_2_. Confluent human FLS or HIG-82 cells were trypsinized with 1:3 dilution of 0.5% trypsin-EDTA (Invitrogen) in Hanks' balanced salt solution. Cellular cytotoxicity was evaluated by monitoring the release of lactate dehydrogenase (LDH) by using a colorimetric detection kit (Roche Diagnostics, Laval, QC, Canada).

### Peptides and recombinant cytokines

Recombinant human cytokines TNF-α and IL-1β were obtained from eBioscience, Inc (San Diego, CA, USA). IDR-1002 peptide (VQRWLIVWRIRK-NH2) [16] was synthesized by using F-moc chemistry at the Nucleic Acid/Protein Synthesis Unit of University of British Columbia, Vancouver, BC, Canada, and IDR-1 peptide (KSRIVPAIPVSLL-NH2) [15] was obtained from GenScript USA Inc. (Piscataway, NJ, USA). The peptides were resuspended in endotoxin-free water, aliquoted, and stored at -20°C. Based on previous studies demonstrating the anti-inflammatory and anti-infective properties of the IDR peptides, in *in vitro *cell studies and *in vivo *models of various infections models [15,16], and on preliminary dose-titration studies, standard doses were used for IDR-1002 (100 μg/ml) and IDR-1 (200 μg/ml) for all experiments.

### ELISA and multiplex flow cytometry

Tissue culture (TC) supernatants were centrifuged at 1,500 *g *for 7 minutes to obtain cell-free samples, aliquoted, and stored at -20°C until further use. Production of MMP-3 was monitored by using Quantikine human MMP-3 (total) ELISA kit (R&D Systems, Inc. Minneapolis, MN, USA), as per the manufacturer's instructions. Production of IL-1RA was monitored in the TC supernatants by using specific antibody pairs from eBioscience, Inc. The production of chemokines IL-8, RANTES, MIG, MCP-1, IP-10 was determined by using a preconfigured multiplex BDCytometric Bead Array (CBA) human chemokine kit by using the FACS Calibur flow cytometer (BD Biosciences, Mississauga, ON, Canada) as per the manufacturer's instructions. The concentration of the cytokines or chemokines in the TC supernatants was evaluated by establishing a standard curve with serial dilutions of the recombinant human cytokines or chemokines, as required.

### Quantitative real-time (qRT-PCR)

Human FLS were stimulated with either IL-1β (10 ng/ml), IDR-1002, or the combination of IL-1β and IDR-1002, for 2 hours. RNA was isolated by using the Qiagen RNeasy kit as per the manufacturer's instructions. Gene expression was subsequently analyzed with qRT-PCR by using SuperScript III Platinum Two-Step qRT-PCR Kit with SYBR Green (Invitrogen), according to the manufacturer's instructions, in the ABI PRISM 7300 sequence-detection system (Applied Biosystems). Fold changes were calculated by the comparative Ct method [26], after normalization with 18sRNA. The list of primers used is shown in Table 1.

**Table 1:** Summary of primers used for quantitative real-time PCR

Gene	Forward primer	Reverse Primer
IL-1RA	ttggaaggctctgaacctca	ctgaaggcttgcatcttgct
SIGIRR	ctcagagccatgccaggt	cctcagcacctggtcttca
18sRNA	gtaacccgttgaaccccatt	ccatccaatcggtagtagcg

### Quantitative proteomics using isobaric tag for relative and absolute quantitation (iTRAQ)

Amine-modifying iTRAQ reagents multiplex kit (Applied Biosystems) was used for relative quantitation of proteins in human FLS stimulated with IL-1β in the presence and absence of IDR-1002 compared with unstimulated (control) cells. Human FLS (2 × 10^4^/ml) were seeded in a total volume of 3 ml per well in a six-well tissue-culture plate in complete DMEM media containing 10% FCS. The cells were allowed to adhere overnight. The following day, the media was changed to 3 ml complete DMEM containing 1% FCS per well. The cells were either unstimulated or treated with IL-1β (10 ng/ml) in the presence or absence of IDR-1002. The peptide was added 45 min before stimulation with IL-1β. After 24 hours of stimulation, the cells were washed with cold PBS and lysed in 250 μl of buffer containing 10 m*M *Tris-HCl pH 7.5, 150 m*M *NaCl, 2 m*M *EDTA, 1% NP-40, and protease inhibitor cocktail (Sigma-Aldrich), on ice for 30 minutes with intermittent vortexing. Cells were centrifuged at 10,000 *g *for 10 minutes at 4°C. Total protein content was estimated in each cell lysate by using micro BCA assay (Pierce; Thermo Scientific, Rockford, IL, USA) with a bovine serum albumin (Sigma-Aldrich) standard curve. The samples were acetone precipitated at -20°C overnight. Proteins were dissolved in 20 μl of iTRAQ dissolution buffer (Applied Biosystems) and further processed as per the manufacturer's instructions. In brief, proteins were reduced and the cysteines blocked by using the reagents in the kit, followed by digestion of the protein samples with provided trypsin solution overnight at 37°C. The trypsin-digested protein samples were labelled with the iTRAQ isobaric tags as follows: unstimulated (control) sample was labeled with iTRAQ isobaric tag 115; IL-1β-stimulated sample, with tag 116; and the isobaric tag 117 was used for labeling the sample obtained from cells treated with IL-1β in the presence of IDR-1002. The contents from each of the iTRAQ reagent-labeled sample was combined together in 1:1 ratio and processed for nanoflow liquid chromatography coupled to tandem mass spectrometry (LC-MS/MS) by using a QStar Elite mass spectrometer (ABSciex, Toronto, ON, Canada).

### Monitoring activation of NF-κB

Rabbit synoviocyte HIG-82 cells were transiently transfected with pNFκB-MetLuc2-Reporter Vector (Clontech Laboratories Inc., Mountain View, CA, USA) or the provided control vector as per the manufacturer's instructions. Various stimulants were added to the transfected cells in culture media containing 1% (vol/vol) FBS. The cells were stimulated with recombinant human IL-1β in the presence and absence of IDR-peptides, either IDR-1002 or IDR-1, for 6 hours. The peptides were added at the time of cytokine stimulation. The activation of NF-κB was monitored by using the Ready-To-Glow Secreted NF-κB Luciferase Reporter Assay (Clontech) as per the manufacturer's instructions.

Human OA FLS were stimulated with IL-1β in the presence and absence of IDR-peptides, IDR-1002 and IDR-1. The peptides were added either 45 minutes before, or at the time of cytokine stimulation. Nuclear extracts were prepared by using NE-PER extraction reagents (Thermo Fisher Scientific) as per the manufacturer's instructions. Nuclear extracts (5 μg) were resolved on 4% to 12% NuPAGE Bis-Tris gels (Invitrogen) and probed with antibodies specific for either NF-κB subunit p50 (Cell Signaling Technology) or antibody to β-actin (Thermo Fisher Scientific) by using immunoblots.

### Monitoring functional JNK activity

Human FLS (5 × 10^4^/ml) were seeded in a total volume of 20 ml per 75-cm^2 ^tissue-culture flask in complete DMEM media containing 10% (vol/vol) FBS for each condition. The cells were allowed to adhere overnight. The next day, the media was changed to 10 ml complete DMEM containing 1% (vol/vol) FBS. The cells were either unstimulated or treated with IL-1β (10 ng/ml) in the presence or absence of IDR-1002 for 15 minutes. IL-1β is known to induce JNK activation after 15 minutes in human FLSs [27]. Total protein concentration was evaluated for each cell lysate by using micro BCA (Thermo Scientific). Kinase activity specific to JNK was monitored by using the JNK activity assay kit (Abcam Inc.) as per the manufacturer's instructions. In brief, 20 μg of total protein per cell lysate was used for immunoprecipitation (IP) by using a JNK-specific antibody. The eluate was treated with c-Jun substrate and ATP mixture. Subsequent phosphorylation of the c-Jun substrate was evaluated by probing immunoblots with anti-phospho-c-Jun (Ser73)-specific antibody.

### Monitoring p38 MAPK activity

Human FLS (5 × 10^4^/ml) were seeded in a total volume of 20 ml per 75-cm^2 ^tissue culture flask in complete DMEM media containing 10% (vol/vol) FBSs for each condition, allowed to adhere overnight, followed by changing the media to 1% (vol/vol) FBS. The cells were either unstimulated or treated with IL-1β (10 ng/ml) in the presence or absence of either IDR-1002 or IDR-1 for 15 minutes. The peptides were added either (a) 45 minutes before cytokine stimulation, or (b) simultaneous with cytokine stimulation. The p38 MAPK activation has been demonstrated in human FLSs on stimulation with IL-1β for 15 minutes [28]. Total protein concentration was evaluated for each cell lysate by using micro BCA (Thermo Scientific). Kinase activity specific to p38 MAPK was monitored by using the p38 MAPK activity assay kit (Cell Signaling Technology) as per the manufacturer's instructions. In brief, 10 μg of total protein per cell extract was used for IP by using a p38 MAPK-specific monoclonal antibody. Kinase activity was evaluated by treating the IP eluates in the presence of ATP and kinase substrate ATF-2 fusion protein. Phosphorylation of the substrate ATF-2 was monitored with Western blot by using a phospho-ATF-2 (Thr76) antibody.

### Immunoblots

The IP eluates or nuclear extracts were electrophoretically resolved on a 4% to 12% NuPAGE Bis-Tris gels (Invitrogen Corporation), followed by transfer to nitrocellulose membranes (Millipore). The nitrocellulose membranes were blocked with TBST (20 m*M *Tris pH 7.5, 150 m*M *NaCl, 0.1% Tween 20) containing 5% (vol/vol) skimmed milk powder. Affinity-purified HRP-linked anti-rabbit secondary antibody was used for detection. The membranes were developed with Amersham ECL detection system (GE Healthcare, Baie d'Urfe, QC, Canada) according to the manufacturer's instructions.

### Microscopy

A modified IDR-1002 was synthesized by incorporating a C-terminal cysteine (IDR-1002C) to allow the presence of a thiol group for biotinylation of the peptide. IDR-1002C was biotinylated, as previously described [29]. In brief, IDR-1002C was biotinylated by using desthiobiotin polyethyleneoxide iodoacetamide (Sigma) as per the manufacturer's instructions. Biotinylated IDR-1002 peptide (IDR-1002B) was purified by HPLC and confirmed by using MALDI mass spectrometry. To facilitate monitoring cellular uptake of the peptide, 150 μl of human FLS (2 × 10^4^/ml) was seeded in 96-well glass-bottom Nunc plates in DMEM containing 10% (vol/vol) FBS, overnight. The following day, the media was changed to DMEM containing 1% (vol/vol) FBS. The cells were stimulated with IDR-1002B (100 μg/ml) for 0, 15, or 30 min. The cells were fixed by using 2% (vol/vol) *para-*formaldehyde, the reaction quenched with 10 m*M *ethanolamine and permeabilized with 0.1% Triton × 100. The cells were washed in PBS and blocked with 3% (vol/vol) FBS in PBS. The cells were stained for actin by using Alexa Fluor 546 phalloidin (Invitrogen) and stained with Streptavidin Alexa Fluor 488 conjugate (Invitrogen) to detect biotin. The cells were counterstained with Hoescht 33258 (Invitrogen) for nuclear staining.

## Results

### IDR-1002 suppressed IL-1β-induced MMP-3, but induced negative regulators of IL-1β in human FLS

**Figure 1:**
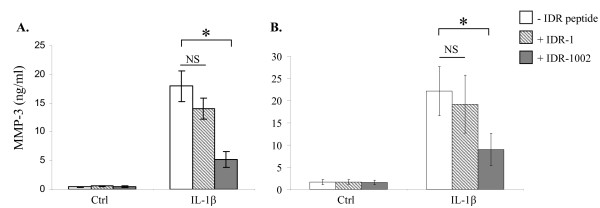
**Evaluation of MMP-3 production in OA and RA FLS**. Human fibroblast-like synoviocytes (FLS) isolated from either **(a)**, osteoarthritis (OA), or **(b)**, rheumatoid arthritis (RA) synovial tissues were stimulated with pro-inflammatory cytokine IL-1β (10 ng/ml) in the presence and absence of IDR peptides. Tissue-culture supernatants were monitored for MMP-3 production by ELISA after 24-hour stimulation. Results shown are an average of at least three independent biologic experiments performed with cells isolated from synovial tissues obtained from independent donors ± standard error (*P < 0.05). IDR, innate defence regulator; IL, interleukin; MMP-3, matrix metalloproteinase-3.

IL-1β induces the production of MMP-3 in FLS, which contributes to the destruction of cartilage and bone in arthritic joints [21]. We therefore evaluated the impact of IDR peptides on IL-1β-induced MMP-3 production. Human FLS isolated from OA and RA synovial tissues were stimulated with IL-1β (10 ng/ml) in the presence and absence of IDR peptides. The peptides were added at the time of cytokine stimulation. The peptides were not cytotoxic to the FLS under any experimental condition, as determined by monitoring the TC supernatants for the release of LDH after 24 hours of stimulation (data not shown). TC supernatants were monitored after 24 hours of stimulation for MMP-3 production by ELISA. IL-1β-induced MMP-3 production was quantitatively similar in OA and RA FLS (Figure 1). IL-1β-induced MMP-3 was significantly suppressed by 70% ± 8% (*P *< 0.05) in the presence of IDR-1002 in OA FLS (Figure 1a). IL-1β-induced MMP-3 was also significantly suppressed by 61% ± 14% (*P *< 0.05) in the presence of IDR-1002 in RA FLS (Figure 1b). In contrast, IDR-1 [15] did not significantly suppress IL-1β-induced MMP-3 production in either OA or RA FLS (Figure 1). Previous studies have shown that human primary OA FLS after stimulation with a pro-inflammatory cytokine such as IL-1β, upregulates the expression of MMP-3, MMP-13, MMP-1, various chemokines, activates transcription factor NF-κB, induces the phosphorylation of ERK, p38, and JNK MAPK, and phosphorylation of AKT, all critical in the induction of inflammatory responses [30,31]. Therefore, we proceeded to investigate further the cellular responses and molecular mechanism of IDR-1002 by using human OA FLS stimulated with pro-inflammatory cytokines. Our approach was consistent with other studies that have used similar *ex vivo *methods with FLSs from OA tissues to investigate potential therapeutic targets and molecular mechanisms of candidate therapeutics for anti-inflammatory interventions [31,32].

**Figure 2:**
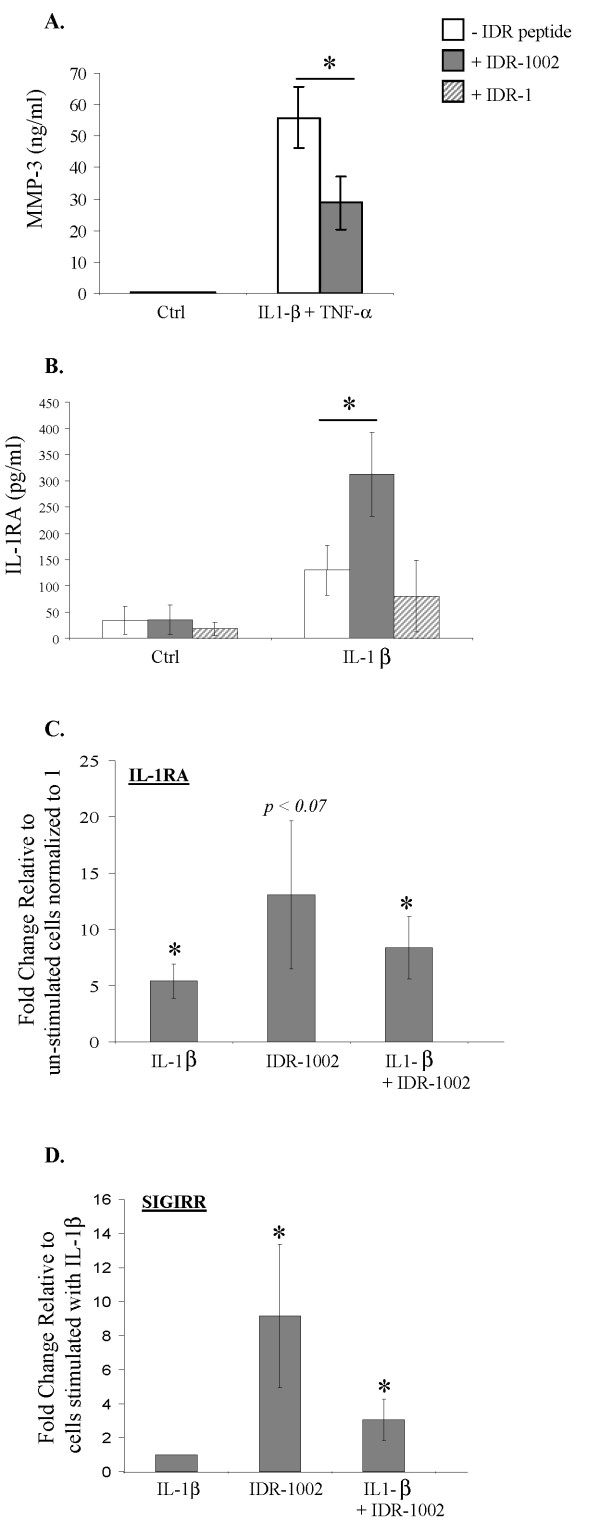
**Evaluation of MMP-3 and IL-1RA production, and transcriptional response of IL-1RA and SIGIRR**. Human fibroblast-like synoviocytes (FLS) were stimulated with pro-inflammatory cytokines either IL-1β (10 ng/ml) or a combination of IL-1β and TNF-α (10 ng/ml each), in the presence and absence of IDR peptides. The peptides were added at the time of cytokine stimulation. Tissue-culture supernatants were monitored for **(a)**, MMP-3 production after 24-hour stimulation, or **(b)**, IL-1RA after 48 hours, with ELISA. Transcriptional responses for **(c)**, IL-1RA, and **(d)**, SIGIRR, were evaluated with quantitative real-time PCR in human FLS cells stimulated with IL-1β (10 ng/ml) in the presence and absence of IDR-1002 after 2 hours. Results shown are an average of at least three independent biologic experiments performed with cells isolated from synovial tissues obtained from independent donors ± standard error (*P < 0.05). IDR, innate defence regulator; IL, interleukin; MMP-3, matrix metalloproteinase-3.

Further to investigate cellular responses in the presence of IDR-1002, human OA FLS were stimulated with pro-inflammatory cytokines, and the TC supernatant was monitored for MMP-3 production after 24 hours and IL-1RA production after 48 hours. MMP-3 production was increased by threefold on stimulation of FLS with cytomix, a combination of 10 ng/ml each of IL-1β and TNF-α (Figure 2a), compared with IL-1β alone (Figure 1a). This elevated level of cytomix-induced MMP-3 was also suppressed by 56% ± 10% (*P *< 0.05) by the peptide IDR-1002 (Figure 2a). IDR-1002 by itself did not induce MMP-3 production above the background amount observed in unstimulated control FLS (Figures 1 and 2a). In contrast, IDR-1002 synergistically enhanced the production of IL-1β-induced IL-1RA by threefold, which was not seen with IDR-1 peptide (Figure 2b). Consistent with this, on monitoring gene expression of endogenous inhibitors of IL-1β [33], it was demonstrated that IDR-1002 by itself induced the gene expression of IL-1RA by > 10-fold, and did not suppress the IL-1β-induced gene expression of IL-1RA (Figure 2c). Similarly, gene expression of another negative regulator of IL-1β, SIGIRR (single Ig IL-1R related molecule, also known as TIR8) was induced more than ninefold (*P *< 0.05) by the peptide IDR-1002 relative to that observed in cells stimulated with IL-1β (Figure 2d). Taken together, these results indicated that IDR-1002 suppressed IL-1β-induced pro-inflammatory MMP-3 production and in contrast enhanced the expression of negative regulators of IL-1β, in human FLS.

### IDR-1002 altered the IL-1β-induced proteome in human FLS

**Figure 3:**
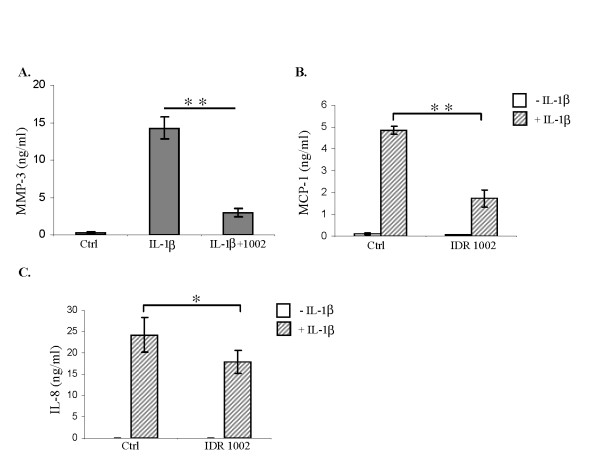
**Monitoring IL-1β-induced MMP-3 and chemokine production on pretreatment with IDR-1002**. Human fibroblast-like synoviocytes (FLS) were pretreated with IDR-1002 for 45 minutes before stimulation with IL-1β (10 ng/ml) for 24 hours. Tissue-culture supernatants were monitored for **(a) **MMP-3 production with ELISA, and chemokines, **(b) **MCP-1, and **(c)**, IL-8 production by using the BDCytometric Bead Array (CBA) preconfigured human chemokine multiplex system with FACS Caliburflow cytometer (BD Biosciences). Results shown are an average of three independent biologic experiments performed with cells isolated from synovial tissues of three different donors ± standard error (*P < 0.05, **P < 0.01). IDR, innate defence regulator; IL, interleukin; MMP-3, matrix metalloproteinase-3.

As an approach to globally defining the impact of IDR-1002 on IL-1β-induced protein production, we undertook a quantitative proteomic analysis. Human OA FLS were pretreated with IDR-1002 45 min before IL-1β (10 ng/ml) stimulation for 24 hours. The TC supernatants were monitored for MMP-3 and chemokine production. IDR-1002 significantly (*P *< 0.01) suppressed IL-1β-induced MMP-3 production by 80% (Figure 3a) and suppressed chemokine MCP-1 production by > 60% (Figure 3b) after 24 hours. However, IL-1β-induced neutrophil chemokine IL-8 production (Figure 3c) was only modestly suppressed (by 20%, *P *< 0.05) by the peptide. These observations were consistent with previous studies demonstrating that HDP can selectively suppress inflammation without abrogating certain chemokine responses that are required for cell movement and recruitment essential to combat infectious assault [3].

The FLS lysates were labeled with iTRAQ reagents; isobaric tag 115 for unstimulated (control) samples, tag 116 for IL-1β-stimulated cells, and the tag 117 for cells treated with the combination of IL-1β and IDR-1002. Samples from three independent donors were individually examined by LC-MS/MS. The mass spectrometry data were analyzed by using ProteinPilot (Applied Biosystems). Only those proteins that were identified in at least two of the three independent experiments with 95% confidence were selected for further analysis. In total, 517 proteins were identified from at least two of the three independent samples stimulated with IL-1β. Proteins were defined to be induced if the relative ratios compared with the unstimulated controls (fold change) were at least mean ± 1.3 standard deviations (which meant that 20% of the population was differentially expressed). Forty-eight of the 517 proteins were defined to be induced on stimulation with IL-1β. Of these 48 IL-1β-induced proteins, 11 proteins were found to be suppressed by IDR-1002 between 20% and 60% (Additional file 1, Table s1). To define immunity-related pathways that may be involved in the alteration of IL-1β-induced responses in the presence of IDR-1002, we undertook a network-based approach. The eleven IL-1β-induced protein candidates that were suppressed by IDR-1002 (Additional file 1, Table S1) were submitted to InnateDB biomolecular interaction database, which facilitates systems-level analysis of mammalian immune genes and protein products [34].

The computational network analysis demonstrated that several members of NF-κB and the mitogen-activated protein kinase-8 (MAPK8) pathways were direct interactors of the selected protein candidates (Additional file 1, Table S2). Four of the 11 selected proteins participated in interactions with candidates known to participate in NF-κB activation, including (i) IκBκE; which activates NF-κB via TRAF-2 [35], (ii) TRAF-6; an NF-κB regulator that plays a critical role in human autoimmune diseases including arthritis [36], (iii) TNF-receptor superfamily member TNFRSF21; which activates NF-κB and MAPK8 pathways [37,38], and (iv) mitogen-activated protein kinase kinase kinase-14 (MAP3K14), also called the NF-kappa-beta-inducing kinase (NIK); which activates NF-κB via TRAF-2 [39]. Several members of the c-Jun N-terminal kinases of the JNK pathway [40], MAPK8, MAPK8IP1, and TNFRSF21, were also identified in the interaction protein network of IL-1β-induced candidates that were suppressed by IDR-1002 in human FLS cells (Additional file 1, Table S2). Another interesting observation from this computational analysis was that genes encoding for four of the 11 selected protein candidates had binding sites for the transcription factor hepatocyte nuclear factor (HNF)-4α (Additional file 1, Table S2).

### IDR-1002 inhibited IL-1β-induced activation of JNK and p38 MAPK

**Figure 4:**
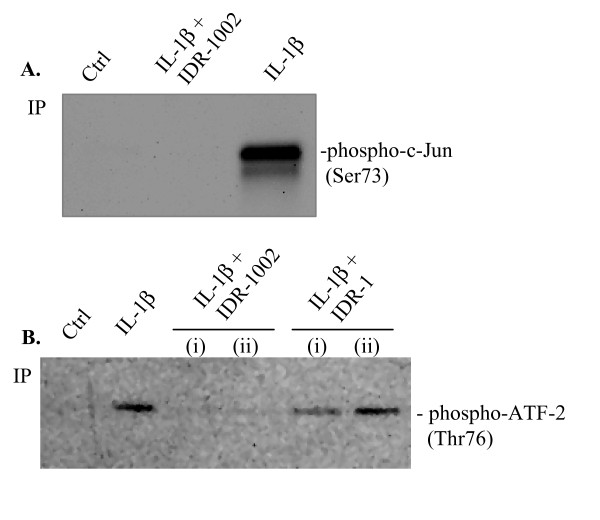
**Evaluation of JNK and p38 MAPK activation**. **(a) **Human fibroblast-like synoviocytes (FLS) were stimulated with IL-1β (10 ng/ml) in the presence and absence of IDR-1002 for 15 minutes. Immunoprecipitation (IP) was performed by using 20 μg of cell lysates with a JNK-specific antibody. The IP eluates were incubated with c-Jun substrate and ATP mixture, and the kinase activity specific to JNK was monitored by monitoring the phosphorylation of the substrate by using an anti-phospho-c-Jun (Ser73)-specific antibody. **(b) **Human FLS were stimulated with IL-1β (10 ng/ml) in the presence and absence of either IDR-1002 or IDR-1 for 15 minutes. The peptides were added either (i) 45 minutes before or (ii) at the time of cytokine stimulation. IP was done by using 10 μg of cell lysates with a p38-specific antibody, and the IP eluates were incubated with substrate ATF-2 protein and ATP mixture. Kinase activity specific to p38 MAPK was evaluated in immunoblots probing with a phospho-ATF-2 (Thr76)-specific antibody. The immunoblots shown are representative of three independent experiments performed with cells isolated from synovial tissues of three different donors. IDR, innate defence regulator; IL, interleukin; JNK, c-Jun N-terminal kinase; MAPK, mitogen-activated protein kinase; MMP-3, matrix metalloproteinase-3.

The network-based interrogation of the proteomics data indicated that members of the JNK pathways (for example, MAPK8, MAPK8IP1) were modulated by the peptide IDR-1002. Therefore, we evaluated the impact of IDR-1002 on IL-1β-induced activation of JNK in human FLS. To monitor JNK activation, human OA FLS were treated with IL-1β in the presence and absence of IDR-1002 for 15 minutes, followed by IP by using a JNK-specific antibody. The IP eluates were treated with c-Jun substrate in the presence of ATP. Phosphorylation of the c-Jun substrate was evaluated with an anti-phospho-c-Jun (Ser73)-specific antibody as a measure of JNK activity. We reproducibly demonstrated that IL-1β-induced JNK activation was abrogated in the presence of IDR-1002 in human FLS (Figure 4a).

Another member of the MAPK family, p38 MAPK, plays a critical role in inflammatory diseases, including in RA [41,42]. Also, p38 MAPK has been demonstrated to be involved in the immunomodulatory mechanism of both IDR peptides, IDR-1 and IDR-1002 [15,16]. Therefore, we evaluated the impact of both these peptides (that is, IDR-1002 and IDR-1) on IL-1β-induced p38 MAPK activation in human FLS. IL-1β activates p38 MAPK in FLS after 15 minutes of stimulation [28]. Human OA FLS were stimulated with IL-1β in the presence and absence of the IDR peptides for 15 minutes. p38 MAPK activity was evaluated by using IP with p38 MAPK (Thr180/Tyr182) monoclonal antibody. The IP eluates were treated with p38 MAPK substrate ATF-2 in the presence of ATP. Phosphorylation of the substrate was monitored in immunoblots, probing with a phospho-ATF-2 (Thr76)-specific antibody as a measure of p38 MAPK activity. We conclusively demonstrated that IDR-1002 abrogated IL-1β-induced p38 MAPK activity in human FLS, whereas peptide IDR-1 had a limited effect (Figure 4b).

### IDR-1002 inhibited IL-1β-induced activation of NF-κB

**Figure 5:**
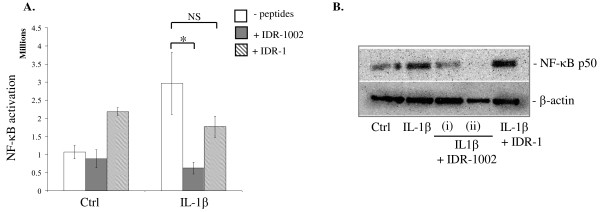
**Monitoring activation of NF-κB**. **(a) **Rabbit synovial fibroblast HIG-82 cells were transiently transfected with pNFκB-MetLuc2-Reporter Vector (Clontech). The transfected cells were stimulated with IL-1β (10 ng/ml), in the presence and absence of either IDR-1002 or IDR-1. The activation of NF-κB was monitored after 6 hours of stimulation by using Ready-To-Glow Secreted NF-κB Luciferase Reporter Assay (Clontech) as per the manufacturer's instructions. Results shown are an average of at least three independent experiments ± standard error (*P < 0.05; NS, nonsignificant). **(b) **Nuclear extracts of human fibroblast-like synoviocytes (FLS) stimulated with IL-1β (10 ng/ml) in the presence and absence of either IDR-1002 or IDR-1 were probed with NF-κB p50 antibody or β-actin antibody in immunoblots. IDR-1002 was added either (i) 45 minutes before, or (ii) at the time of cytokine stimulation. Result shown is a representative blot of three independent experiments performed with FLS obtained from three different donors. IDR, innate defence regulator; IL, interleukin; NF-κB, nuclear factor-kappaB.

The network-based interrogation of the proteomics data showed that members of the NF-κB pathway were altered by the peptide IDR-1002. Therefore, to confirm the proteomics data, we evaluated IL-1β-induced activation of NF-κB in the presence and absence of IDR-1002 in synovial fibroblasts. To monitor NF-κB direct activation, a rabbit synovial fibroblast cell line (HIG82) was transiently transfected with pNFκB-MetLuc2-Reporter Vector (Clontech). The cells were stimulated with IL-1β (10 ng/ml each), in the presence and absence of either IDR-1002 or IDR-1. The activation of NF-κB was monitored after 6 hours of stimulation by using the Ready-To-Glow Secreted NF-κB Luciferase Reporter Assay (Clontech) as per the manufacturer's instructions. Peptide IDR-1 by itself activated NF-κB, which was not observed by the peptide IDR-1002 (Figure 5a). IL-1β-induced NF-κB activation was significantly (*P *< 0.05) neutralized by IDR-1002 (the levels were below those observed in control unstimulated cells), but not by the peptide IDR-1 (Figure 5a).

We also monitored NF-κB activation in primary human OA FLS by evaluating the nuclear translocation of NF-κB subunit p50 after stimulation with IL-1β in the presence and absence of IDR-peptides. IDR-1002 significantly suppressed IL-1β-induced nuclear translocation of NF-κB p50, whereas IDR-1 did not (Figure 5b).

### IDR-1002 was internalized in human FLS

**Figure 6:**
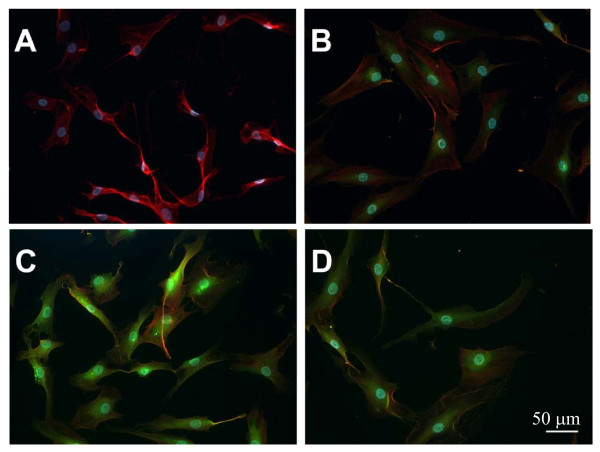
**Evaluating cellular uptake of IDR-1002**. Human fibroblast-like synoviocytes (FLS) were stained fluorescently to demonstrate the uptake of IDR-1002. The cells were stimulated with 100 μg/ml biotinylated IDR-1002 (IDR-1002B). Cells were either **(a)**, not treated with peptide, or stimulated with IDR-1002B for **(b)**, 0 minutes, **(c)**, 15 minutes, or **(d)**, 30 minutes. Cells were washed, fixed, and stained for actin by using Alexa Fluor 546 phalloidin (red) and stained with Streptavidin Alexa Fluor 488 conjugate to detect IDR-1002B (green). Hoescht 33258 was used to counterstain the nucleus (blue). The scale bar in **(d) **represents a length of 50 μm. IDR, innate defence regulator.

Previous studies demonstrated cellular uptake of cationic HDP in monocytic cells, dendritic cells, and epithelial cells, and implicated that inhibition of cellular uptake/endocytic mobilization interferes with cellular responses mediated by these peptides [43-45]. In this study, we wanted to monitor whether the peptide IDR-1002 was internalized in human FLS. Recent studies have demonstrated cellular uptake of HDP and IDR peptides by using biotinylated peptides and have shown that C-terminal cysteine modification and biotinylation does not alter the immune-modulatory activity of these peptides [29,43]. Consistent with this, intracellular receptors have been recently demonstrated for HDP LL-37 and IDR-1 [29,43]. Therefore, we used a biotinylated IDR-1002 (IDR-1002B) to monitor cellular uptake in human OA FLS after 15 and 30 minutes of stimulation. These time points were selected because we demonstrated that IDR-1002 altered IL-1β-induced cell signaling after 15 minutes of stimulation (Figure 4). In this study, we showed that IDR-1002B was effectively taken up in human FLS after 15 minutes of stimulation and that the cellular localization was largely cytosolic (Figure 6).

## Discussion

In this study, we examined the use of innate immune-modulatory IDR peptides in limiting IL-1β-induced inflammatory responses in synovial fibroblasts. We demonstrated that a 12-amino-acid IDR peptide, IDR-1002 [16], controlled IL-1β-induced inflammatory responses in synovial fibroblasts largely by intervening with IL-1β-induced NF-κB, JNK, and p38 MAPK activation. A recent study demonstrated the ability of IDR-1002 to protect against bacterial infections largely by modulating host immune responses [16]. A distinct advantage of the class of IDR peptides is the likelihood that these agents can control inflammation while maintaining elements of innate immunity required for efficient anti-infective mechanisms [3,14,15,46], which is speculated to be distinct from current anti-TNF therapies.

In this study, we showed that IDR-1002 significantly suppressed IL-1β-induced MMP-3 and MCP-1 protein production in FLS. MMP-3 (stromelysin 1) is known to be elevated in both OA and RA, and it promotes the destruction of matrix components of the joints [21]. MCP-1 is a monocyte chemoattractant highly expressed in the synovial fluid and tissues of RA patients [47]. Production of MCP-1, either by macrophages or by FLS, results in an autocrine or paracrine stimulation of cells within the synovial microenvironment, resulting in overall extracellular matrix degradation [48]. For example, MCP-1 increases collagenase activity and induces MMP-3 release from chondrocytes [48]. Therefore, significant suppression of IL-1β-induced production of both MMP-3 and MCP-1 in human FLS demonstrates the therapeutic potential of IDR-1002.

We showed that even though IDR-1002 significantly suppressed IL-1β-induced MMP-3 production (Figures 1 and 3a), and chemokine MCP-1 production (Figure 3b), the peptide did not significantly suppress the expression of an anti-infective neutrophil chemokine (that is, IL-8 production in human FLS; Figure 3c). In contrast, IDR-1002 induced gene expression of endogenous inhibitor of IL-1β (for example, IL-1RA (Figure 2c) and enhanced IL-1β-induced production of IL-1RA protein (Figure 2b) in human FLS). It should be noted that recombinant IL-1RA (anakinra) has been extensively explored as a potential therapy for RA [33]. This selective and differential modulation of inflammatory responses by the IDR-peptide is consistent with previous studies. For example, both HDP and IDR peptides can modestly induce classic pro-inflammatory responses, such as certain chemokine production in macrophages required for anti-infective immunity [3,15,16]. In contrast, these peptides significantly induce anti-inflammatory mediators such as IL-10 [3,15,16]. These peptides result in a net balancing of inflammation. In this study, we showed such "selective" modulation of cytokine-mediated inflammatory response by IDR-1002 in synovial fibroblasts. Thus, based on previous study [16] and this study, we can speculate that IDR-1002 can exhibit a targeted immune-modulatory activity on both immune cells (for example, macrophages [16]), and mesenchymal cells, such as FLS.

We demonstrated that IDR-1002 modulated IL-1β-induced responses in synovial fibroblasts primarily by intervening with the activation of JNK and p38 MAPK (Figure 4) and NF-κB (Figure 5) signaling pathways. In this study, it was demonstrated that IDR-1002 suppressed IL-1β-induced proteins that are regulated by the JNK and NF-κB pathways (Additional file 1, Table S2). Consistent with this, by using immunochemical assays, we showed that IDR-1002 abrogated both IL-1β-induced JNK and p38 MAPK activity in human FLS (Figure 4), and significantly suppressed the IL-1β-induced activation of NF-κB in synovial fibroblasts (Figure 5). These results are consistent with previous studies that have demonstrated that both HDP and IDR-peptides can influence MAPK pathways and selectively modulate pathogen-induced NF-κB regulation [3,15,16,49]. IL-1β-induced JNK and p38 MAPK are critical in the induction of MMPs and subsequent tissue destruction in arthritis [42,50]. Consequently, both JNK and p38 MAPK are defined as a valuable therapeutic targets for arthritis [51-53]. It should be noted that HDP (for example, the human cathelicidin LL-37) by itself can transiently and differentially activate the NF-κB subunits [49]. However, in this study, IDR-1002 did not induce NF-κB activity by itself in synovial fibroblasts at the time point monitored (Figure 5a). Taken together, this is consistent with the paradigm of "selective" immunomodulation of inflammatory responses by HDP and IDR-peptides (that is, suppression of excessive activation of NF-κB in the presence of exogenous infectious/inflammatory stimuli, while maintaining transient NF-κB activity), overall resulting in a net balanced inflammation.

An interesting observation from the computational analysis of the proteomics data in this study was the implication of the involvement of transcription factor HNF-4α-targets in IDR-1002-mediated anti-inflammatory activity (Additional file 1, Table S2). A publication in 2009 by Chenomx Inc. describing the analysis of serologic metabolite profiles in RA, demonstrated the interconnectedness between the IL-1β-induced inflammatory protein networks and the transcription factor HNF-4α-mediated signaling. The modulation of HNF-4α-target elements by IDR peptides warrants further investigation [54].

In this study we also demonstrated cellular uptake and cytosolic localization of IDR-1002 in human FLS (Figure 6). Cellular uptake and endocytic mobilization of cationic HDP has been shown in monocytic cells and epithelial cells, and it was previously suggested that cellular uptake may be essential for immune-modulatory activity for cationic peptides [43,44]. Even though some intracellular interacting protein partners and putative cell-surface receptors have been identified for both human HDP LL-37 and IDR-1 [29,43,55], the mechanism of receptor interaction for IDR peptides is yet to be completely elucidated. A recent study suggested that IDR-1002 activity may be mediated by a Gi-coupled receptor [16]; however, no direct interacting protein partner or receptor has been defined for IDR-1002. Taken together, based on previous studies and this study, we can speculate the action of IDR-1002 on FLS as follows. IDR-1002 may be internalized by FLS either interacting with putative cell surface receptors or, more likely, by inserting directly into the cell membrane, as proposed by previous studies for cationic peptides [45,56]. A vesicle-mediated uptake pathway, as previously implicated for HDP [44,45,56], is likely to facilitate the internalization of IDR-1002, and subsequent interaction with putative intracellular interacting protein partners or receptors, as described for other HDP and IDR peptides [29,43]. Identification of intracellular receptors for IDR-1002 in mesenchymal cells such as human FLS warrants further investigation and is beyond the scope of this article. Interaction of the peptide with putative intracellular protein partners may be facilitating alteration of innate immune signaling pathways, overall resulting in the modulation of inflammatory responses by IDR-1002 in synovial fibroblasts. Results from this study provide evidence that supports further research into the development of IDR-peptides as potential therapeutics in immune-mediated chronic inflammatory diseases such as RA.

## Conclusions

This study demonstrates that an IDR peptide, based on naturally occurring innate immune effector HDP, can selectively modulate IL-1β-induced responses in synovial fibroblasts. This study showed that IDR-1002 peptide suppressed IL-1β-induced inflammatory responses by altering the IL-1β-induced proteome, suppressed IL-1β-mediated activation of NF-κB, MAPK p38, and JNK, suppressed IL-1β-induced MMP-3 and MCP-1 production, but did not neutralize the production of all chemokines that are required for resolution of infections. In contrast, the peptide enhanced the expression of anti-inflammatory endogenous inhibitors of IL-1β (for example, IL-1RA). Overall, this study showed that an IDR-peptide can selectively modulate immune-mediated "sterile" inflammation in mesenchymal cells such as FLS. This study provides a new potential application of innate immune IDR-peptides as selective immune-modulatory agents that are likely to control inflammation without hampering anti-infective immunity. We propose that IDR-peptides represent valuable candidates that should be explored further as potential therapeutics for inflammatory arthritis and other diseases characterized by chronic inflammation.

## Abbreviations

FLS: fibroblast-like synoviocytes; HDP: host defense peptide; IDR: innate defence regulator; JNK: c-Jun N-terminal kinases; MAPK: mitogen-activated protein kinase; MCP-1: monocyte chemotactic protein-1; MMP-3: matrix metalloproteinase-3; NF-κB: nuclear factor kappa-B; OA: osteoarthritis; RA: rheumatoid arthritis.

## Competing interests

The authors declare that they have no competing interests.

## Authors' contributions

ETB performed all experiments, except proteomics and microscopy, and contributed to writing the manuscript. KGC performed all experiments, except proteomics and microscopy. DNDL performed the microscopy experiments. JPC performed the computational analysis for the proteomics data. REWH provided the IDR-1002 peptide and edited the manuscript. HEG provided tissues for isolation of human FLS, provided intellectual input, and edited the manuscript. NM conceived the study, performed the proteomics iTRAQ experiments, and wrote the manuscript. All authors contributed to the conception and/or acquisition of data and analysis for this project, and to either drafting or revising the manuscript.

## Supplementary Material

Additional file 1 **Supplementary Table 1. IL-1β-induced proteins suppressed in the presence of IDR peptide, IDR-1002**. Human fibroblast-like synoviocytes (FLS) were stimulated with IL-1β (10 ng/ml) in the presence and absence of IDR-1002 for 24 hours. The peptide was added 45 minutes before cytokine stimulation. The cell lysates were processed for iTRAQ labelling by using three different isobaric tags. Three independent LC-MS/MS runs were performed on iTRAQ-labelled samples from three independent donors. Protein candidates were selected only if they were detected in at least two of the three independent biologic experiments. Eleven proteins induced by IL-1β were found to be suppressed by IDR-1002 between 20% and 60%. **Supplementary Table 2. Computational network-based analysis by using InnateDB biomolecular network database**. IL-1β-induced protein candidates that were found to be suppressed between 20% and 60% by IDR-1002 (11 proteins) were submitted to InnateDB biomolecular interaction database [57]. This database was used to identify direct interactions between the selected 11 protein candidates and any known immunity-related proteins. The identified interactions are summarized, and the members of NF-κB and JNK pathways, and association of HNF-transcription factor, are indicated in bold in this table. Click here for file
