# Supplementary material for: Modulation of interleukin-1β-induced inflammatory responses by a synthetic cationic innate defence regulator peptide, IDR-1002, in synovial fibroblasts
Source: Arthritis Res Ther. 2011 Aug 11;13(4):R129. doi: 10.1186/ar3440 (PMC3239371; doi:10.1186/ar3440)
Supplement: Additional file 1 — Supplementary Table 1. IL-1β-induced proteins suppressed in the presence of IDR peptide, IDR-1002. Human fibroblast-like synoviocytes (FLS) were stimulated with IL-1β (10 ng/ml) in the presence and absence of IDR-1002 for 24 hours. The peptide was added 45 minutes before cytokine stimulation. The cell lysates were processed for iTRAQ labelling by using three different isobaric tags. Three independent LC-MS/MS runs were performed on iTRAQ-labelled samples from three independent donors. Protein candidates were selected only if they were detected in at least two of the three independent biologic experiments. Eleven proteins induced by IL-1β were found to be suppressed by IDR-1002 between 20% and 60%. Supplementary Table 2. Computational network-based analysis by using InnateDB biomolecular network database. IL-1β-induced protein candidates that were found to be suppressed between 20% and 60% by IDR-1002 (11 proteins) were submitted to InnateDB biomolecular interaction database [57]. This database was used to identify direct interactions between the selected 11 protein candidates and any known immunity-related proteins. The identified interactions are summarized, and the members of NF-κB and JNK pathways, and association of HNF-transcription factor, are indicated in bold in this table. [file ar3440-S1.PDF]

Supplementary Information:

Supplementary Table 1: ***IL-1β-induced proteins suppressed in the presence of IDR peptide, IDR-1002.*** Human FLS were stimulated with IL-1β (10 ng/ml) in the presence and absence of IDR-1002 for 24 hr. The peptide was added 45 min prior to cytokine stimulation. The cell lysates were processed for iTRAQ labelling using three different isobaric tags. Three independent LC-MS/MS runs were performed on iTRAQ-labelled samples from three independent donors. Protein candidates were selected only if they were detected in at least two out of the three independent biological experiments. 11 proteins induced by IL-1β were found to be suppressed by IDR-1002 between 20-60%.

**Gene Name**	**REFSEQ_ PROTEIN**	**Suppression of IL-1β-induced protein in the presence of IDR-1002**
Adenylosuccinate synthase (ADSS)	NP_001117	60 %
Ribosomal protein L27A (RPL27A)	NP_000981	42 %
Kynureninase / L-Kynurenine hydrolase (KYNU)	NP_001028170	38 %
Aldo-keto reductase family 1, Member C3 (AKR1C3)	NP_003730	38 %
Amyloid beta (A4) precursor protein (APP)	NP_958817	31 %
Aminopeptidase-like-1 (NPEPL1)	NP_078939	27 %
Annexin A5 (ANXA5)	NP_001145	24 %
Carboxylesterase 1 (CES1) / Monocyte macrophage serine esterase 1	NP_001020365	24 %
Aldo-keto reductase family 1, Member B1 (AKR1B1)	NP_001619	23 %
Cytochrome P450, family 1, subfamily B, polypeptide 1 (CYP1B1)	NP_000095	23 %
Actin, beta (ACTB)	NP_001092	21 %

Supplementary Table 2: ***Computational network-based analysis using InnateDB biomolecular network database.*** IL-1β-induced protein candidates that were found to be suppressed between 20 – 60 % by IDR-1002 (11 proteins) were submitted to InnateDB biomolecular interaction database[1]. This database was used to identify direct interactions between the selected 11 protein candidates and any known immunity-related proteins. The identified interactions are summarized, and the members of NF-κB and JNK pathways, and association of HNF-transcription factor, are indicated in bold in this table.

**RefSeq**	**Name**	**Interaction**	**Interaction Type**	**PMID**
NP_001117	ADSS	ADSS interacts with **IKBKE** and with HLA-B	physical association	17353931
NP_001117	ADSS	Transcription factor **HNF4A** binds with ADSS gene	unspecified	14988562
NP_078939	NPEPL1	No interactions		
NP_000981	RPL27A	RPL27A interacts with **MAP3K14**	physical association	14743216
NP_000981	RPL27A	ACTB::ADSL;TNRC6B, AGK, ARF4, ATP5C1, ATP5I, CCT5, CDIPT, DBT, DNAJA1, DNAJA2, DNAJB11, EEF1A1, EIF2C2, EIF2C3, EIF2C4, EIF4B, EMD, GALK1, HIST1H2AB, HNRNPC, HSP90AA1, HSP90AB1, HSPA1B, HSPA5, HSPA8, IDBG-12906, IGF2BP1, IPO8, JAK1, MYCBP, PABPC1, PABPC4, PGAM5, PRDX1, PRMT5, PTGES3, PTS, QPCTL, RBM10, RPL11, RPL12, RPL23, RPL24, RPL27, RPL27A, RPL35, RPL38, RPL8, RPS10L, RPS12, RPS18, RPS25, RPS26, RPS3A, RPS5, RPS9, SLC25A1, SLC25A10, SLC25A13, SLC25A22, SLC25A3, SLC25A5, SNRPD2, SSBP1, SUCLA2, TNRC6A, TRIM21, TUBA1A, TUBB, TUBB2C, TUFM, TUT1, WDR77, YBX1 (complex)	unspecified	19167051
NP_000981	RPL27A	ATP5C1::C3orf26, COPA, DDOST, DDX20, DDX39, DHCR7, DHX15, DHX30, DHX36, DHX9, EEF1A1, EIF2C2, EPRS, FBL, GEMIN4, GNL3, HNRNPC, HNRNPU, HRNR, ILF2, MRPS22, MRPS27, PABPC1, PHB, PHB2, PRMT5, PTCD3, RBM10, RNF149, RPL11, RPL13A, RPL23A, RPL24, RPL26, RPL27A, RPL27A, RPL29, RPL3, RPL31, RPL35, RPL4, RPL6, RPL8, RPS15A, RPS18, RPS2, RPS26P25, RPS3, RPS3A, RPS4X, RPS5, RPS6, RPS8, RPS9, RUVBL2, SF3B1, SF3B2, SF3B3, SLC25A3, SLC25A6, SLC4A5, SNORD58B, SYNE1, UBA52, UBA52, XRCC6, YBX1 (complex)	unspecified	17932509
NP_001028170	KYNU	No interactions		
NP_003730	AKR1C3	AKR1C3 interacts with MAGEA11, RIF1, AC1N1, C1orf103	physical association	16189514|16169070
NP_003730	AKR1C3	AKR1C3 interacts with MAGEA11, ZHX1, UBE2W	unspecified	16189514|16169070
NP_003730	AKR1C3	Transcription factor **HNF1A** binds with AKR1C3 gene	unspecified	14988562
NP_958817	APP	Cleavage reaction involving APP and CTSD	cleavage reaction	17112520
NP_958817	APP	Cleavage reaction involving APP and CASP3; CASP6; CASP8	cleavage reaction	10409650

[1] Lynn DJ, et al (2008). InnateDB: facilitating systems-level analyses of the mammalian immune response. Mol Syst Biol. 4:218

NP_958817	APP	Cleavage reaction involving ADAM17 and APP	cleavage reaction	9774383
NP_958817	APP	Colocalization of APP and CHRNA7	colocalization	10681545
NP_958817	APP	Colocalization of APP and APP	colocalization	16286452
NP_958817	APP	Colocalization of APP and **MAPK8IP1**	colocalization	11517249
NP_958817	APP	Colocalization of APP and MAPT	colocalization	16446437
NP_958817	APP	Colocalization of APP and PIN1	colocalization	16554819
NP_958817	APP	Colocalization of APP and BACE1	colocalization	10531052
NP_958817	APP	APP, **MAPK8, MAPK8IP1** (complex)<br>APP, **MAP3K11, MAPK8IP1** (complex)	complex assembly	12917434
NP_958817	APP	APP (complex)	direct interaction	18805418
NP_958817	APP	APP interacts with **TNFRSF21**, MAPT, NGFR	direct interaction	16446437| 19225519
NP_958817	APP	Phosphorylation of APP by **MAPK8**	phosphorylation	12917434
NP_958817	APP	Phosphorylation of APP by Abl1	phosphorylation reaction	11279131
NP_958817	APP	APP, GSK3A, MAPT (complex)	phosphorylation reaction	16446437
NP_958817	APP	APP interacts with APOA1, APBB1, APBB2, APBB3, APBA1, SHC1, SHC3, **TGFB2**, **TGFB1**, CHRNA7, TP53BP2, **MAPK8IP1**, PRNP, HSD17B10, GRB2, APOE, ACHE, TTR, A2M, FLOT1, Slc5a7, PSEN1, PSEN2, NF1, PDIA3, PIN1, TUBB, NSF, STXBP1, DNM1, DNAH1, HSP90AA1, HSPA8, CRYAB, PPIA, SPTAN1, ACTB, NEFL, MBP, GFAP, YWHAZ, UCHL1, PGAM1, **MAP3K5,** SMUG1	physical association	11297421|16049941| 9461550|8887653| 12485888|11877420| 8855266|2119582| 10681545|11278849| 11724784|11517249| 16286452|9338779| 9461550|10428074| 9325095|9889152| 8700886|8915609| 9501253| 16480949|17709753|9223340| 16374483|19225519|15896298| 16554819|
NP_958817	APP	APP physically interacts with **MAPK8IP1**	physical interaction	12917434|11724784| 11517249
NP_958817	APP	APP physically interacts with XIAP	physical interaction	14576775
NP_958817	APP	APP interacts with KLK6	protein cleavage	12878203
NP_958817	APP	APP interacts with BACE1	protein cleavage	10531052
NP_000095	CYP1B1	CYP1B1 interacts with SAE1	physical association	17353931
NP_000095	CYP1B1	Transcription factor **HNF4A** binds with CYP1B1 gene	unspecified	14988562
NP_001145	ANXA5	ANXA5 interacts with FDFT1, SUPT4H1, EIF4G1, CFTR, IFNGR2	physical association	16169070
NP_001145	ANXA5	ANXA5 interacts with ITGB5	unspecified	12769841
NP_001145	ANXA5	Transcription factor **HNF4A** binds with ANXA5 gene	unspecified	14988562
NP_001020365	CES1	CES1 interacts with CES1, GUSB	unspecified	12679808| 10562416

NP_001619	AKR1B1	AKR1B1 interacts with I**KBKE, TRAF6,** HLA-B, SMAD1, TFE3, DSP, MCC, DST, PAX7. CSMD1, ZNF253, VHL	physical association	17353931| 15231748
NP_001619	AKR1B1	Transcription factor **HNF4A** binds with AKR1B1 gene	unspecified	14988562
NP_001092	ACTB	A2M::ACTB, ALPP, APOD, ARL8B, ASAH1, ATP5A1, ATP5B, ATP6V0D1, ATP6V1A, ATP6V1B2, AZU1, CAPN6, CKMT1A, CTSG, CYP11A1, CYP19A1, DDOST, DLST, GAPDH, GBA, GLB1, GUSB, HSPA5, HSPD1, MAOA, MPO, PRTN3, SCARB2, SLC25A5, SLC25A6, STS, TPP1, VDAC1 (complex)	colocalization	17174955
NP_001092	ACTB	Colocalization of ACTB and IDBG-44570	colocalization	17525734
NP_001092	ACTB	Colocalization of ACTB and RP23-157O10.7	colocalization	17525734
NP_001092	ACTB	Colocalization of ACTB and FBL	colocalization	16514417
NP_001092	ACTB	Colocalization of ACTB and Pkd1	colocalization	17707375
NP_001092	ACTB	Colocalization of ACTB and MMP14	colocalization	18164686
NP_001092	ACTB	Colocalization of ACTB and BCAR1	colocalization	18164686
NP_001092	ACTB	ACTB and Sorbs1	direct interaction	17082770
NP_001092	ACTB	ACTB and NCF1C	direct interaction	16375898
NP_001092	ACTB	ACTB (complex)	direct interaction	18234857
NP_001092	ACTB	ACTB interacts with CFL1, CFL2, ACTB, ACTG1, DSTN, AR	physical association	16189514| 17353003
NP_001092	ACTB	ACTB::ANXA1, ANXA2, ANXA6, ATP5A1, CD4, DDX3X, DDX5, DHRS2, EEF1B2, EIF3B, EIF3C, EIF3D, EIF3E, EIF3I, EIF3K, EIF3M, EIF4A2, ENO1, FARSB, GAPDH, GNAI2, GNB2L1, HIST1H2BI, HNRNPA1, HNRNPA2B1, HNRNPD, HNRNPH1, HSP90AA1, HSP90AB1, HSPA8, HSPD1, KPNB1, LCK, LRPPRC, MME, MYL12A, MYL6, NCL, NPM1, PHB, RAN, RPL11, RPL18, RPL22, RPL7, RPL7A, RPLP0, RPLP1, RPS10, RPS12, RPS13, RPS18, RPS19, RPS24, RPS3A, RPS4X, RPS7, RPS8, RPS9, RPSA, SSRP1, TNPO1, TUBA1A, TUBB, UBC, VDAC1, VDAC2, VDAC3, VIM, YBX1 (complex)	physical association	15047060
NP_001092	ACTB	ACTB interacts with SMAD3, SMAD9, MDM2, NSMAF, ATF7IP, TJP1, YWHAZ, BBS1, BBS4, APP	physical association	15527767| 17373842| 17599063| 15231748| 16944923| 15161933| 16049941
NP_001092	ACTB	ACTB physically interacts with TSC1	physical interaction	17355907
NP_001092	ACTB	ACTB::ACTL6A, KAT5, RUVBL1, RUVBL2, TRRAP (complex)	unspecified	10966108
NP_001092	ACTB	ACLY::ACTB, ACTG1, ACTN4, CDK6, CDKN2A, EEF2, EPHA3, GAPDH, HNRNPA2B1, HNRNPC, HSP90AA1, HSP90AB1, HSPA4, HSPA8, HSPA9, MCM6, MMRN1, MTR, MYL12A, PCNA, PDCD6;AHRR, RIN2, RUVBL2, SNRPA, SNRPB, TUBA1A, TUBA1C, TUBB, TUBB2C, UBE4B, USP26 (complex)	unspecified	17955473
